# Diagnostic capability of a linear discriminant function applied to a novel Spectralis OCT glaucoma-detection protocol

**DOI:** 10.1186/s12886-020-1322-8

**Published:** 2020-01-29

**Authors:** Maria P. Bambo, Enrique Fuentemilla, Beatriz Cameo, Isabel Fuertes, Blanca Ferrandez, Noemi Güerri, Vicente Polo, Jose M. Larrosa, Luis E. Pablo, Elena Garcia-Martin

**Affiliations:** 1 Ophthalmology Department, Miguel Servet University Hospital, Zaragoza, Spain; 2 Aragon Institute for Health Research (IIS Aragon). Miguel Servet Ophthalmology Innovation and Research Group (GIMSO), Zaragoza, Spain. University of Zaragoza, C/ Padre Arrupe. Consultas Externas de Oftalmología, 50009 Zaragoza, Spain

**Keywords:** Linear discriminant function, Optical coherence tomography, Glaucoma

## Abstract

**Background:**

Bruch membrane opening–minimum rim width (BMO–MRW) assessment offers a new diagnostic use in glaucoma patients of the Glaucoma Module Premium Edition (GMPE) available for the Spectralis optical coherence tomography (OCT) system. The objective of our research was to evaluate the diagnostic benefits of examining BMO–MRW and peripapillary retinal nerve fibre layer (pRNFL) readings acquired with Spectralis OCT to distinguish between healthy and mild glaucoma patients, comparing those readings with the standard pRNFL application. Moreover, we investigated whether using a particular combination of BMO–MRW and pRNFL parameters with a linear discriminant function (LDF) could further enhance glaucoma diagnosis.

**Methods:**

One hundred thirty-six eyes from 136 individuals were incorporated into this observational, prospective cross-sectional study: 68 mild primary open-angle glaucoma (POAG) patients according to the Hodapp-Parrish-Anderson criteria (mean deviation between 0 and − 6 dB) and 68 healthy control subjects selected by Propensity Score Matching. MRW and pRNFL thickness around the disc (diameters: 3.5 mm, 4.1 mm, and 4.7 mm) were obtained using the BMO–MRW protocol, and pRNFL thickness at 3.5 mm was obtained with the standard glaucoma application. The group data were contrasted. One sample was chosen at random to develop the LDF (teaching set: 34 healthy subjects and 34 POAG patients) using a combination of MRW and pRNFL parameters (acquired with the BMO–MRW protocol); the other sample provided a test of how the LDF performed on an independent group (validating set: 34 healthy subjects and 34 POAG patients). The receiver operating curves (ROCs) were plotted for every measurement and contrasted with the proposed LDF. The OCT parameters with the best area under the receiver operating characteristic curve (AUC) were determined.

**Results:**

Global MRW and pRNFL thicknesses were significantly thinner in the POAG group (*p* <  0.001). The BMO–MRW parameters showed good diagnostic accuracy; the largest AUCs reached 0.875 for the LDF and 0.879 for global RNFL thickness using the standard glaucoma application. There were no statistical differences between the AUCs calculated.

**Conclusions:**

BMO–MRW parameters show a strong capability to differentiate between mild glaucoma and control eyes. Our LDF based on the new BMO–MRW OCT protocol did not perform better than isolated parameters.

## Background

Spectral domain optical coherence tomography (SD-OCT) has furthered anatomical understanding of the optic nerve structures, enhancing delimitation of the optic nerve head (ONH) margin [1, 2]. Most commercially available structural diagnostic tools used to detect glaucoma are based on evaluation of the ONH and the retinal nerve fibre layer (RNFL). In contrast, the Glaucoma Module Premium Edition (GMPE) of the Spectralis version 6.0 SD-OCT software (Heidelberg Engineering, Inc), which includes an anatomic positioning system (APS), accurately determines neuroretinal rim tissue by measuring the minimum distance between the Bruch membrane opening (BMO) and the internal limiting membrane (ILM). This BMO–minimum rim width (MRW) provides the most geometrically accurate measurement of the neuroretinal rim [3, 4].

Recent studies have demonstrated higher glaucoma diagnosis accuracy using BMO–MRW and a stronger association with functional parameters compared to conventional rim parameters [5–7]. Various authors have indicated the utility of BMO–MRW in diagnosing glaucoma in moderate myopic eyes [8] or small optic discs [9]. BMO–MRW also showed high intraday repeatability, similar to the RNFL thickness parameters, and independence from intraindividual intraocular pressure (IOP) changes [10].

Given the significance of this new parameter, the study goal was to assess the diagnostic capability of BMO–MRW analysis and peripapillary RNFL (pRNFL) evaluation using three circles (diameters: 3.5 mm, 4.1 mm and 4.7 mm) acquired with SD-OCT APS software. The study contrasted healthy subjects against mild glaucoma patients and compared the results with those obtained using standard pRNFL evaluation. We also checked whether using a specific combination of BMO–MRW and pRNFL parameters in conjunction with a linear discriminant function (LDF) could further enhance glaucoma diagnosis [11]. In addition, this is the first paper to ascertain the diagnostic capability of an LDF designed specifically for the Spectralis OCT platform and based entirely on results obtained with the novel BMO–MRW method.

## Methods

Some of the procedures detailed in this paper were described in Bambo MP, et al. [12].

### Study population and design

In this observational, prospective cross-sectional study, subjects were enrolled sequentially from the Glaucoma Department at the Miguel Servet University Hospital (Zaragoza, Spain). All participants in the research were advised of its nature and potential results and subsequently gave their written informed consent. The study protocol adhered to the tenets of the Declaration of Helsinki and was approved by the Regional Clinical Ethics Committee of Aragón (CEICA). The control group was populated with healthy age- and sex-matched individuals (mostly hospital workers) who came for a spectacle prescription, contact lens fitting, etc. and were examined by our department.

Based on a preliminary study conducted by our group, the sample size required to detect differences of at least 4 μm in the RNFL thickness measured by OCT was calculated, applying a bilateral test with risk α of 5% and risk β of 10% (i.e. with a power of 90%). In order to obtain a sufficient sample of glaucoma patients to allow an in-depth study of the history of the disease, the unexposed/exposed ratio was determined to be 0.5. With these data it was concluded that at least 102 eyes would be necessary (51 from healthy subjects and 51 from glaucoma patients). A total of 68 healthy control eyes and 68 mild primary open-angle glaucoma eyes were included to increase the potency of the study.

One of the requirements for inclusion was prior clinical diagnosis of mild primary open-angle glaucoma (POAG) no fewer than 12 months earlier. The diagnostic criteria comprised characteristic glaucomatous optic nerve damage (either a clear notch in the neuroretinal rim or an increase in cup-to disc ratio) identified using the slit-lamp test, corresponding defects in the visual field (VF), an open anterior chamber angle assessed using gonioscopy, and increased intraocular pressure (IOP, > 21 mmHg). The presence of 3 or more significant (*p* <  0.05) non-edge continuous points (at least 1 of which had to be at the *p* <  0.01 level on the same side of the horizontal meridian in the pattern deviation plot), confirmed on two consecutive VF examinations, was considered a glaucomatous VF defect and therefore “outside normal limits” on the Glaucoma Hemifield Test. Only patients with mild glaucoma per the Hodapp-Parrish-Anderson criteria (mean deviation between 0 and − 6 dB [13]) were selected for this study.

All healthy subjects who enrolled in the study (both teaching and validating populations) had normal optic discs, normal VF readings, IOP ≤ 21 mmHg in both eyes and no history of intraocular disease or surgery or family history of glaucoma.

When recruiting for the POAG and control groups, subjects were rejected if they suffered loss of vision due to another ocular disease, had received laser treatment in the past 2 months or had undergone ocular surgery in the 3 months prior to the study. Similarly, subjects were excluded if they presented extreme refractive errors such as high myopia (− 6.0 or higher), hyperopia (+ 6.0 or higher) or astigmatism (±3.0 or higher) or if they presented evidence of macular, vascular or inflammatory pathologies or non-glaucomatous optic nerve neuropathies. Subjects suffering clinically relevant lenticular opacity as per the LOCS III classification [14] were likewise excluded, taking as the criteria in this instance cortical cataract graded above C2, nuclear colour above NC2, opalescence above NO2 and posterior subcapsular cataract graded greater than or equal to P1. One eye per subject was selected at random.

### Ophthalmologic examination

The visual examinations, performed between September 2017 and March 2018, comprised evaluation of best-corrected visual acuity (Snellen chart at 4 m) and IOP (calibrated Goldmann applanation tonometer), slit-lamp examination of the anterior segment, and fundus assessment. VF was evaluated using the Humphrey 24–2 Swedish Interactive Threshold Algorithm Standard perimeter (Zeiss Meditec, Dublin, CA). The VF reliability parameters were set at < 20% fixation errors and < 33% false positives/negatives and only readings that met those criteria were selected. The VF examinations were conducted within 2 months of taking the OCT measurement.

### Optical coherence tomography measurement

The study eye of all participating subjects was imaged using the new GMPE on the Spectralis software version 6.0 SD-OCT platform (Heidelberg Engineering, Inc). All scans were performed by an experienced system operator (B.C.). Signal quality for all images captured was ≥25 dB. Subject eyes were evaluated using the GMPE software, which includes APS. Placement of the examination ring was automatic and was determined by 2 fixed anatomical landmarks: the centre of the fovea and the centre of the BMO, creating a fovea–BMO centre axis.

**Fig. 1:**
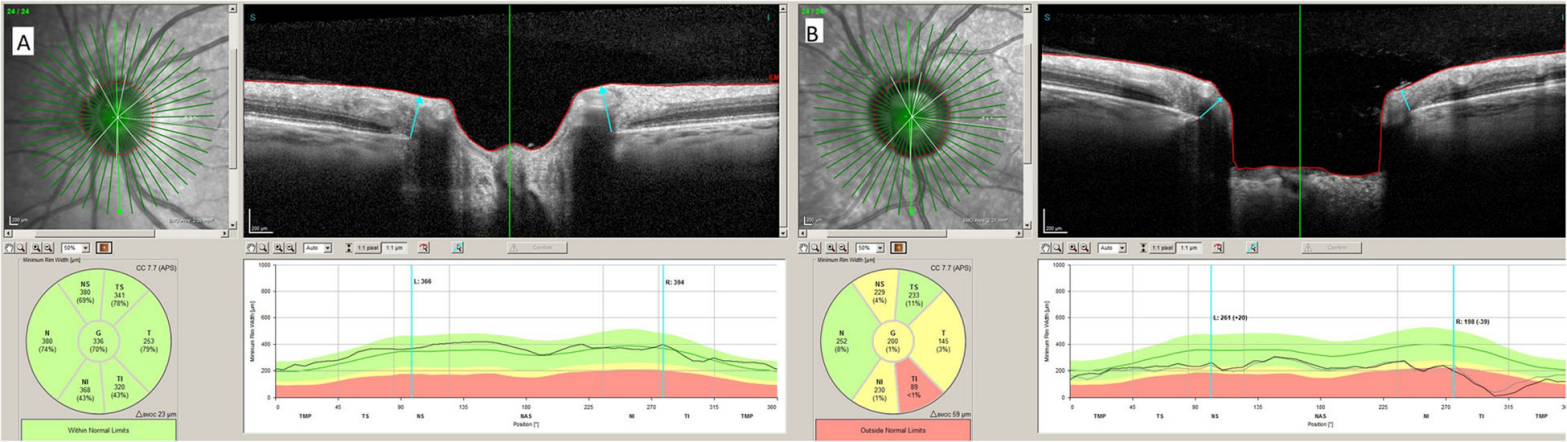
Images of the acquisition in the left eyes of a healthy subject (**a**) and a glaucoma patient (**b**) using the optical nerve head rim analysis possible with Spectralis’s optical coherence tomography Glaucoma Module Premium Edition. The new software performs 24 high-resolution 15° radial scans (green lines in the upper left infrared image). In the upper right B-scan image of each figure, minimum rim width (MRW) (blue arrow) is measured from the Bruch membrane opening (red dots) to the nearest point on the internal limiting membrane (red line). The lower left images in each figure show a representation of MRW by 6 sectors and global MRW thickness with a colour code (green equals within normal limits, yellow equals borderline and red equals outside normal limits). The lower right images represent MRW thickness in a linear graph

**Fig. 2:**
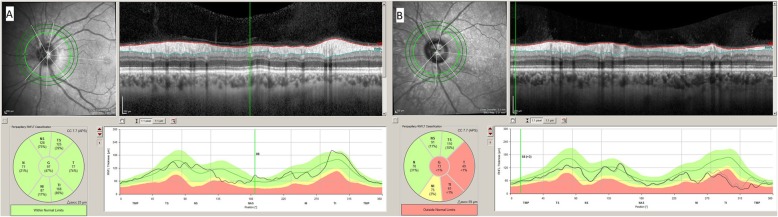
Images of the acquisition in the left eyes of a healthy subject (**a**) and a glaucoma patient (**b**) using the peripapillary retinal nerve fibre layer (pRNFL) analysis possible with Spectralis’s optical coherence tomography Glaucoma Module Premium Edition. pRNFL is measured in three concentric circles (diameters: 3.5 mm, 4.1 mm and 4.7 mm) around the optic nerve head (green lines in the upper left infrared image in each figure). In the upper right B-scan image in each figure, pRNFL thickness at 3.5 mm diameter is measured between the red line (internal limiting membrane) and the blue line (RNFL-ganglion cell layer interphase). The lower left images in each figure show a representation of pRNFL at 3.5 mm by 6 sectors and global pRNFL thickness with a colour code (green equals within normal limits, yellow equals borderline and red equals outside normal limits). The lower right images represent pRNFL thickness at 3.5 mm in a linear graph

Analysis of the ONH rim (24 high-resolution 15° radial scans, each averaged from 27 B-scans) automatically defined the BMO and ILM, which were subsequently corrected manually if necessary. The neuroretinal rim was assessed from the BMO to the nearest point on the ILM [4, 15], producing a shortest-distance measurement known as the BMO–MRW (Fig. 1a and b). This parameter, which quantifies neuroretinal rim tissue perpendicular to axon orientation, takes account, at all measurement points [5], of the trajectories of the nerve fibres entering the ONH. Circular scans using pRNFL GMPE were carried out to obtain RNFL thicknesses around the disc (diameters: 3.5 mm, 4.1 mm, and 4.7 mm) (Fig. 2a and b).

Lastly, we performed a circular scan using the standard pRNFL application (Spectralis OCT Glaucoma, without APS), measuring RNFL thickness around the disc (3.5 mm diameter; 16 averaged consecutive circular B-scans; 768 A-scans).

Eight eyes (5 POAG eyes and 3 control eyes) were excluded due to low OCT scan quality (truncated B-scans in which the internal limiting membrane could not be segmented due to the presence of blood vessels or vitreous detachment, and/or an image quality score < 25 due to keratitis).

### Statistical analysis

All statistical analyses were performed with SPSS (version 20.0; SPSS Inc., Chicago, IL) and MedCalc (MedCalc Software, Mariakerke, Belgium) statistical software. The Kolmogorov-Smirnov test was used to assess sample distribution. The control group was selected by Propensity Score Matching from a sample pool of healthy subjects. A total of 136 subjects (control and glaucoma) participated in the study. One sample was chosen at random to develop the LDF (teaching set) and the other sample was used to check the performance of the LDF in an independent population (validating set). The teaching set was used to analyse binary logistic regression, performed when the dependent variable presents a dichotomy (healthy or diseased) and the independent variables do not. The dependent variable was mild POAG (yes/no) and the predictive variables were the BMO–MRW and pRNFL parameters measured using the new Spectralis OCT GMPE application.

The anticipated dependent variable is therefore a function of the probability that a subject will fall into one of the two categories (e.g., the probability that a subject has mild POAG, based on his/her scores on the predictive variables). The relative significance of each independent variable was evaluated using the Wald stepwise binary logistic regression analysis. The Wald w2-statistic tests the unique contribution made by each predictor in relation to the others (the other predictors remaining constant) and removes predictor overlap.

This means that the parameters with the highest sensitivity/specificity values may not necessarily be those selected in the logistic regression method. The criteria used to determine the variables included in and excluded from the model were set by the stepwise probability test. The LDF was calculated from the weighted sum of the predictor variables. The significant BMO–MRW and pRNFL OCT parameters were then combined to create a new variable (the LDF) to maximize the measurable differences between healthy and mild POAG eyes. The diagnostic accuracy of our LDF was tested by using the validating set to compare it with other isolated OCT parameters generated by Spectralis OCT. To simplify presentation of the results and adopt the most reproducible values [16], when calculating the AUCs we selected only the global or average values of the various OCT parameters. The ROC curves were plotted for all of the parameters and compared with the proposed LDF.

The Hanley-Mc Neal method [17] was used to test differences between the ROC curves so as to compare the AUC. MedCalc software was used to calculate the cut-off points, determined as those with the best sensitivity-specificity balance. Positive and negative likelihood ratios (LRs) were likewise calculated.

The Bonferroni correction was applied when multiple comparisons were made (*p* = 0.05 / number of comparisons).

### Results

A total of 144 subjects were recruited and examined in consecutive order. Of that number, 8 were excluded on the grounds of poor OCT image quality, leaving a final study group of 136 (68 healthy subjects and 68 mild glaucoma patients). A total 68 eyes were analysed as the teaching set, 34 eyes from 34 mild POAG patients and 34 eyes from 34 healthy subjects examined. Mean age was 62.85 ± 8.25 in the POAG group and 65.32 ± 10.48 in the healthy group.

The validating set comprised 34 mild POAG eyes and 34 healthy eyes (not included in the teaching set). In the healthy group, the mean age was 62.32 ± 8.68, while in the POAG group it was 64.20 ± 9.72. There were no significant differences in age, sex or spherical equivalent between the groups in any of the samples.

Visual field parameters showed significant differences between POAG and controls: mean deviation of − 2.82 ± 0.25 in POAG and − 0.31 ± 0.22 in controls (*p* <  0.001), pattern standard deviation (PSD) of 2.91 ± 1.48 in POAG and 1.59 ± 0.40 in controls (*p* <  0.001), visual field index (VFI) of 94.98% ± 5.08 in POAG and 99.17% ± 0.97 in controls (*p* <  0.001).

A summary of the clinical characteristics is set out in Table 1.

**Table 1:** Clinical characteristics and main optical coherence tomography parameters of the glaucoma patients and the healthy subjects

	Teaching Set (*n* = 68)			Validating Set (*n* = 68)		
	Healthy Subjects (*n* = 34)	Glaucoma Group (*n* = 34)		Healthy Subjects (*n* = 34)	Glaucoma Group (*n* = 34)	
	Mean ± SD	Mean ± SD	P*	Mean ± SD	Mean ± SD	P*
Age (years)	62.85 ± 8.25	65.32 ± 10.48	0.055	62.32 ± 8.68	64.20 ± 9.72	0.062
Sex (men:women)	20:14	18:16	0.324	15:19	17:17	0.216
BCVA (Snellen)	0.96 ± 0.08	0.93 ± 0.13	0.469	0.97 ± 0.06	0.91 ± 0.08	0.010
IOP (mm Hg)	17.46 ± 3.10	16.89 ± 0.493	0.493	17.33 ± 2.96	17.30 ± 3.32	0.967
MD (dB)	−0.34 ± 1.45	−2.85 ± 1.97	**< 0.001**	− 0.26 ± 1.47	−2.78 ± 1.71	**< 0.001**
Spherical Equivalent (D)	0.93 ± 1.57	0.35 ± 2.39	0.190	0.82 ± 1.69	0.17 ± 2.24	0.219
CCP (μm)	558.01 ± 41.25	535.14 ± 37.32	0.072	556.33 ± 42.31	550.33 ± 34.55	0.540
pRNFL glaucoma global (μm)	97.96 ± 8.58	80.93 ± 13.94	**< 0.001**	96.44 ± 9.21	76.67 ± 13.88	**< 0.001**
BMO–MRW area (mm^2^)	1.99 ± 0.31	1.96 ± 0.36	0.722	1.98 ± 0.32	1.95 ± 0.39	0.813
BMO–MRW global (μm)	313.03 ± 54.87	236.94 ± 73.65	**< 0.001**	314.81 ± 56.12	227.93 ± 63.85	**< 0.001**
pRNFL 3.5 global (μm)	96.93 ± 7.27	83.65 ± 15.65	**< 0.001**	97.89 ± 8.23	78.79 ± 15.71	**< 0.001**
pRNFL 4.1 global (μm)	83.76 ± 5.88	71.10 ± 12.92	**< 0.001**	84.11 ± 7.58	68.71 ± 14.33	**< 0.001**
pRNFL 4.7 global (μm)	73.25 ± 5.36	63.75 ± 12.25	**< 0.001**	73.74 ± 6.60	60.86 ± 11.90	**< 0.001**

P*: level of statistical significance in comparison between groups using the t–test (except for gender, Chi-square). Bold text indicates statistically significant results with Bonferroni correction (*p* <  0.004)

*SD* Standard deviation, *BCVA* Best-corrected visual acuity, *IOP* Intraocular pressure, *MD* Mean deviation (visual field), *dB* decibels, *D* Dioptres, *CCP* Central corneal pachymetry, *pRNFL* peripapillary retinal nerve fibre layer, *BMO–MRW* Bruch membrane opening–minimum rim width

All global pRNFL parameters significantly decreased in the POAG groups of both samples (teaching and validating). There were significant differences in global BMO–MRW thickness (*p* <  0.001) between groups, but BMO–MRW area was not significantly lower in the POAG group in either the teaching or validating set (Table 1).

Using a stepwise procedure, the BMO–MRW and pRNFL parameters of the novel Spectralis GMPE OCT protocol that produced the greatest error were identified. These were then included in the model, with the next-best variable subsequently being identified and included, and so on. The LDF was thus defined as follows: 37,200 – (0.324 × pRNFL 4.7 mm temporal inferior sector) – (0,197 × pRNFL 4.7 mm temporal superior sector) + (0.541 × pRNFL 4.7 mm temporal sector) – (0.117 × pRNFL 3.5 mm nasal superior sector).

In the validating set, the sensitivity (Se)-specificity (Sp) balances were highest for the LDF (Se: 70.37, Sp: 96.15, cut-off point: > 0.309) and for global pRNFL thickness using the standard glaucoma application (Se: 91.18, Sp: 73.53, cut-off point: ≤ 92). The largest AUCs were 0.875 (95% confidence interval [CI], 0.755–0.950) for the LDF; and 0.879 (95% CI, 0.778–0.946) for global pRNFL thickness using the standard glaucoma application. The other AUCs, the best sensitivity-specificity balance and the likelihood ratios of various OCT parameters in the validating set are summarized in Table 2.

**Table 2:** Validating set: areas under the ROC curves of various optical coherence tomography parameters

	AUC	95% CI	AUC P*	Cut-off Point	Sens (%)	Spec (%)	+LR	-LR
LDF	0.875	0.755–0.950	**< 0.001**	> 0.309	70.37	96.15	18.30	0.31
pRNFL glaucoma global	0.879	0.778–0.946	**< 0.001**	≤ 92	91.18	73.53	3.44	0.12
BMO–MRW global	0.846	0.736–0.923	**< 0.001**	≤ 250.08	75.76	96.97	25	0.25
pRNFL 3.5 global	0.844	0.724–0.927	**< 0.001**	≤ 86	75.86	89.29	7.08	0.27
pRNFL 4.1 global	0.812	0.684–0.905	**< 0.001**	≤ 70	67.86	92.59	9.16	0.35
pRNFL 4.7 global	0.816	0.689–0.908	**< 0.001**	≤ 64	71.43	88.89	6.43	0.32

MedCalc software was used to calculate the cut-off points (points with the best sensitivity-specificity balance)

P*: level of statistical significance (< 0.05). Bold text indicates statistically significant results (*p* < 0.05)

*AUC* Area under the ROC curve, *CI* Confidence interval, *+LR* Positive likelihood ratio, *−LR* Negative likelihood ratio, *BMO–MRW* Bruch membrane opening–minimum rim width, *LDF* Linear discriminant function, *pRNFL* peripapillary retinal nerve fibre layer

There were no statistical differences between the AUCs calculated (Table 3).

**Table 3:** Validating set: differences between the areas under the ROC curves (Hanley-MacNeil method)

	LDF	pRNFL glaucoma global	BMO–MRW global	pRNFL 3.5 global	pRNFL 4.1 global	pRNFL 4.7 global
LDF	____					
pRNFL glaucoma global	0.988	____				
BMO–MRW global	0.471	0.445	____			
pRNFL 3.5 global	0.390	0.157	0.981	____		
pRNFL 4.1 global	0.200	0.026	0.699	0.227	____	
pRNFL 4.7 global	0.185	0.047	0.701	0.274	0.938	____

*BMO–MRW* Bruch membrane opening–minimum rim width, *LDF* Linear discriminant function, *pRNFL* peripapillary retinal nerve fibre layer

statistically significant results with Bonferroni correction (*p* < 0.003)

## Discussion

This study examined the diagnostic capability of BMO–MRW analysis and pRNFL (obtained with three circles; diameters: 3.5 mm, 4.1 mm and 4.7 mm) evaluation using SD-OCT APS software to differentiate healthy subjects from mild glaucoma patients, as compared with the standard pRNFL application (without APS). Previous evaluations of the role of BMO–MRW parameters in glaucoma [18, 19] found a strong diagnostic capability similar to that of the standard pRNFL application and better than that achieved with a confocal scanning laser ophthalmoscope. This study is the first to compare the diagnostic capability of an LDF designed for the Spectralis OCT and based exclusively on results obtained with the novel BMO–MRW protocol. Another of the strengths of this research is its confirmation of the LDF on an independent sample [11]. Our results showed that the OCT parameters obtained with the BMO–MRW and the Spectralis OCT GMPE (either BMO–MRW or pRNFL thickness in three concentric circles cantered on the ONH) have a similar diagnostic capability to the standard pRNFL application in differentiating mild glaucoma eyes from control eyes; and that our LDF, designed with a combination of OCT parameters taken from the new BMO–MRW protocol, performed similarly to isolated OCT parameters as regards diagnosis.

In this study, we found differences between mild glaucoma eyes and control eyes (both BMO–MRW and pRNFL) in all global OCT parameters analysed, except in the case of BMO–MRW area. This contrasts with other authors, such as Enders et al. [20], who found that BMO–MRW area offers good diagnostic capability in a large glaucoma cohort (including different levels of glaucoma and ocular hypertensive patients). This difference could be due to the early stage of glaucoma and the small sample size in our study.

Our LDF, developed from OCT parameters obtained with the novel BMO–MRW application for the Spectralis OCT GMPE platform, did not show better diagnostic accuracy than single global OCT parameters. Surprisingly, our final LDF only included pRNFL measurements, and no BMO–MRW values: 37,200 – (0.324 × pRNFL 4.7 mm temporal inferior sector) – (0.197 × pRNFL 4.7 mm temporal superior sector) + (0.541 × pRNFL 4.7 mm temporal sector) – (0.117 × pRNFL 3.5 mm nasal superior sector). It is important to note that parameters providing higher sensitivity and/or specificity values may not necessarily be selected as logistic regression variables.

Previous studies have shown the clinical utility of BMO–MRW parameters in diagnosing glaucoma, especially in cases of extreme optic-disc sample size, demonstrating better diagnostic capability than confocal scanning laser tomography and exhibiting a good structure function correlation [9, 21]. In general, many studies have shown the SD-OCT BMO–MRW and pRNFL thickness parameters to surpass confocal scanning laser tomography as regards diagnostic capacity to detect glaucoma [3, 5, 18, 20, 22]. There is less agreement on whether preference should be given to one of the two morphometric SD-OCT parameters. Chauhan et al. showed BMO–MRW to outperform pRNFL thickness as regards sensitivity in revealing glaucomatous damage [23]. Gardiner et al. recently showed that pRNFL thickness might outperform BMO–MRW in follow-up assessment of glaucoma patients due to higher correlation to development of visual field defects [22]. The authors of a previous study showed that uncorrected BMO–MRW was influenced by ONH size and was physiologically thinner in healthy eyes, reducing comparability between ONH sizes [24–26]. In consequence, for BMO–MRW, correction of the measurements for ONH size appears to play a crucial role in increasing diagnostic capability to detect glaucoma.

The limitations to this study were the small sample size and the lack of correction of BMO–MRW parameters dependent on papillary size and morphology, which could condition the results obtained. More studies evaluating the influence of small or large discs, myopic discs and tilting of the disc in BMO–MRW parameters are yet to be addressed.

## Conclusions

To summarize, this study did not find differences in diagnostic accuracy between the BMO–MRW and pRNFL parameters (obtained using the new GMPE software and the standard Spectralis SD-OCT Glaucoma application); and our LDF based on the new BMO–MRW OCT application was not superior to isolated parameters. New studies including larger sample sizes which take into account special ONH situations are necessary to elucidate the role of BMO–MRW in glaucoma diagnosis.

## Data Availability

The datasets used and analysed during the current study are available from the corresponding author on reasonable request.

## Abbreviations

APS: Anatomic Positioning System
BMO: Bruch Membrane Opening
CI: Confidence Interval
GMPE: Glaucoma Module Premium Edition
ILM: Internal Limiting Membrane
IOP: Intraocular Pressure
LR: Likelihood Ratios
MRW: Minimum Rim Width
ONH: Optic Nerve Head
POAG: Primary open-angle glaucoma
pRNFL: peripapillary retinal nerve fibre layer
RNFL: Retinal Nerve Fibre Layer
SD-OCT: Spectral Domain Optical Coherence Tomography
Se: Sensitivity
Sp: Specificity
